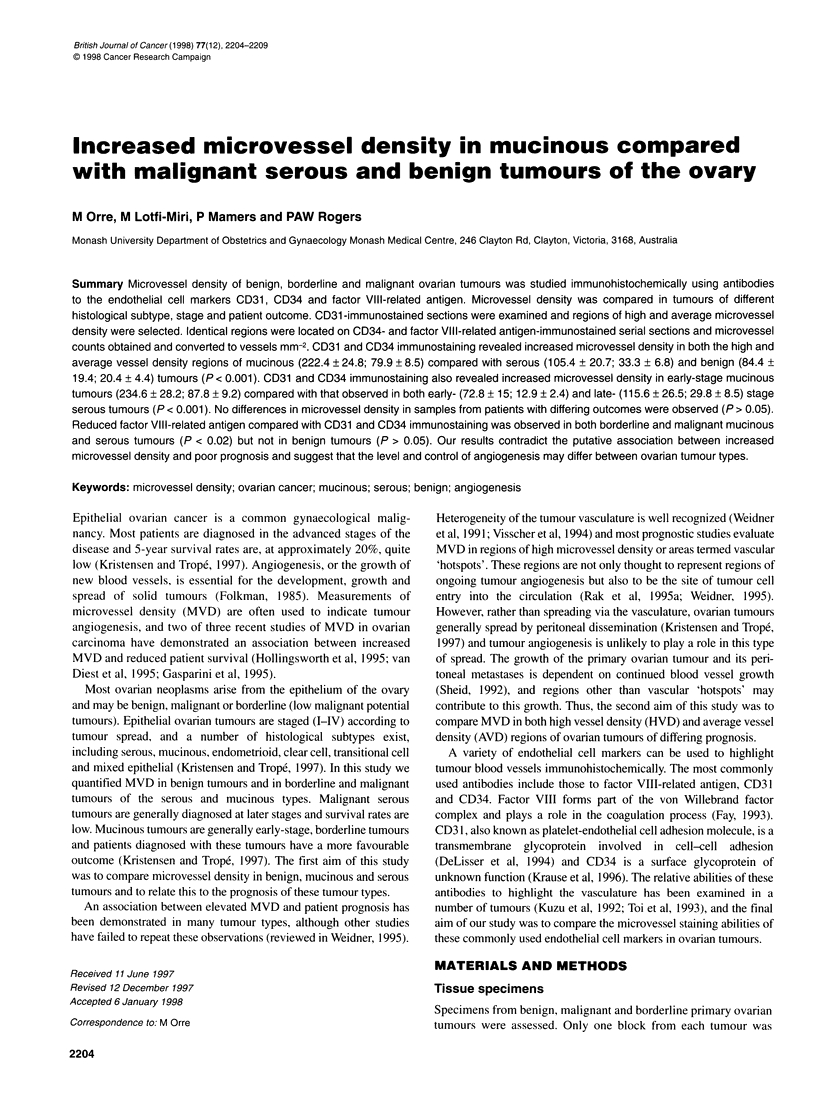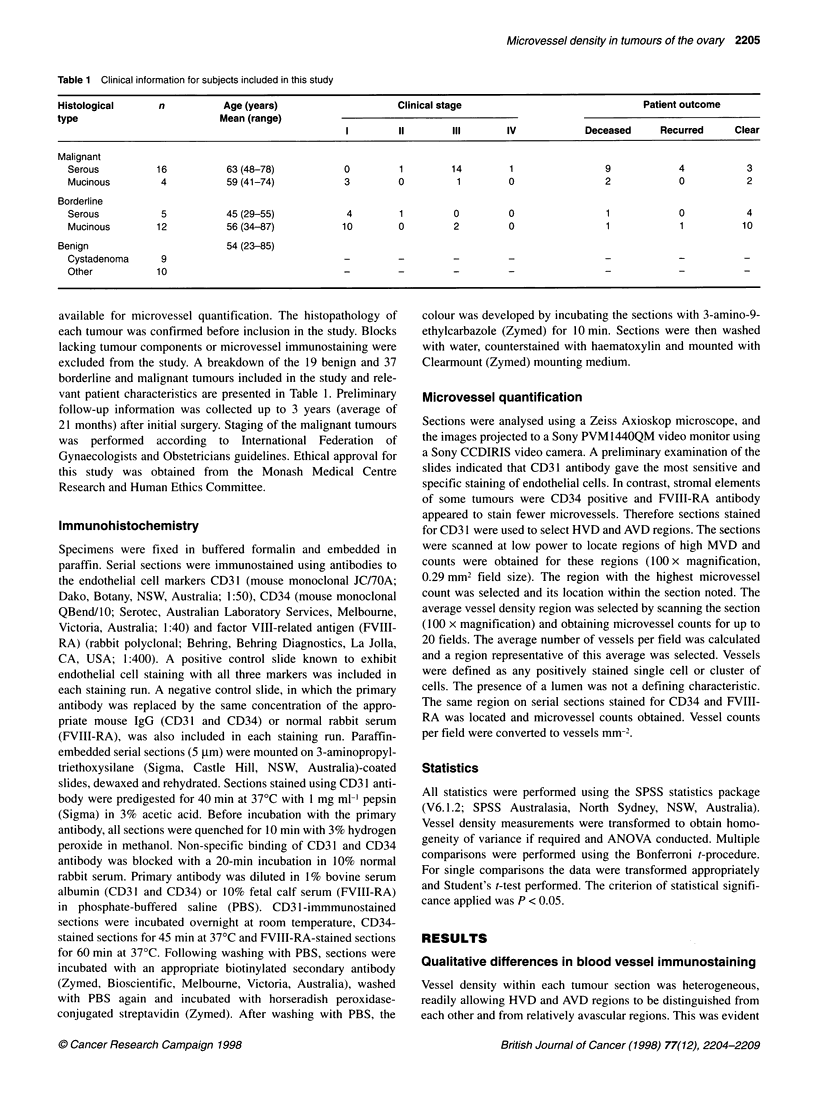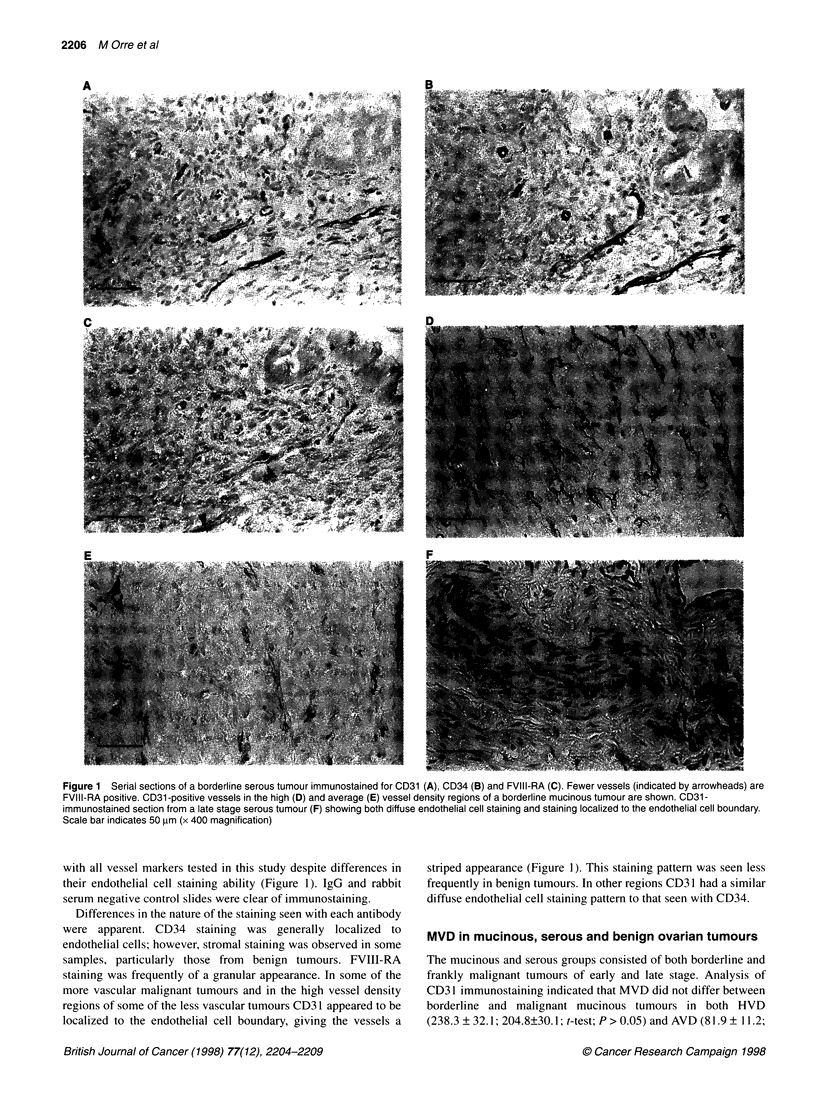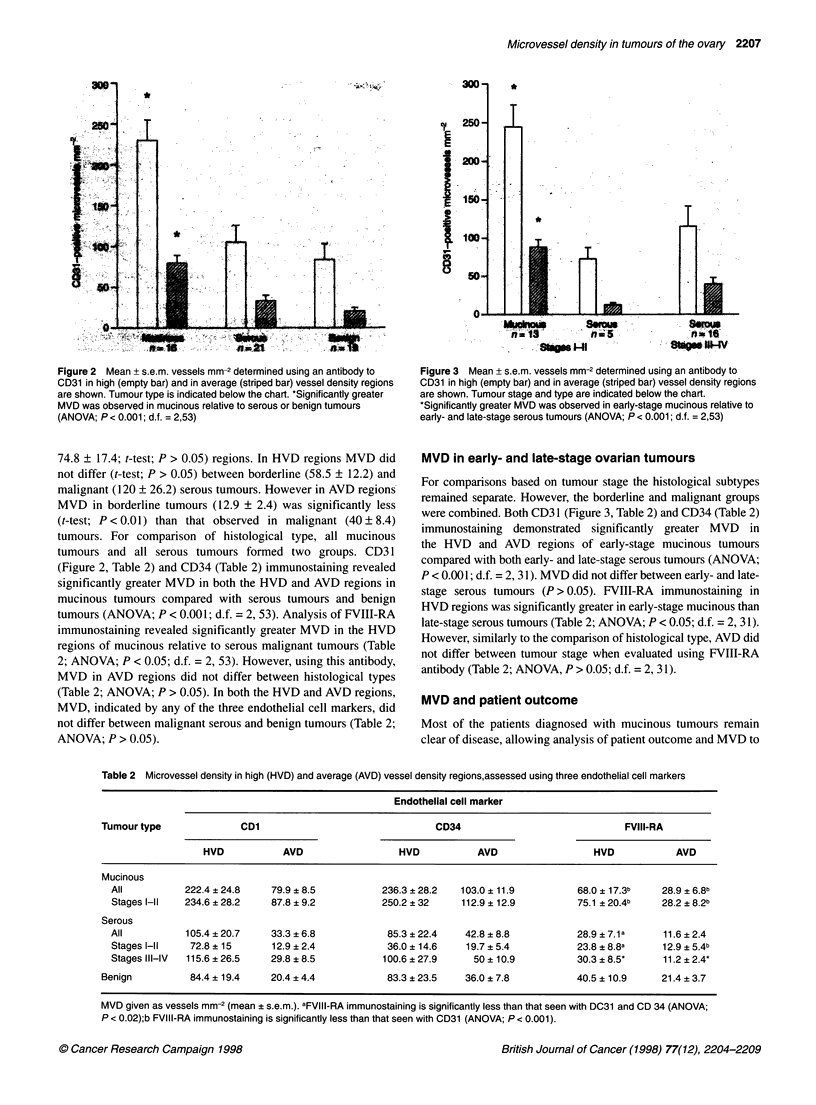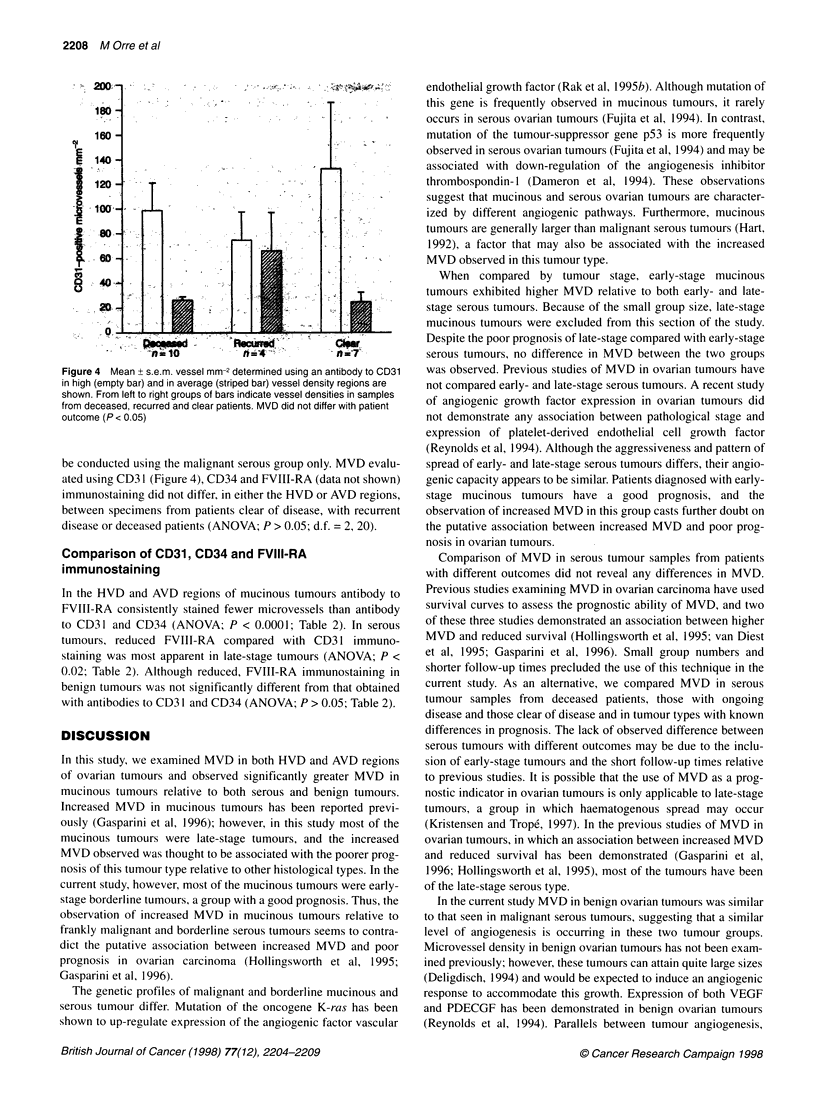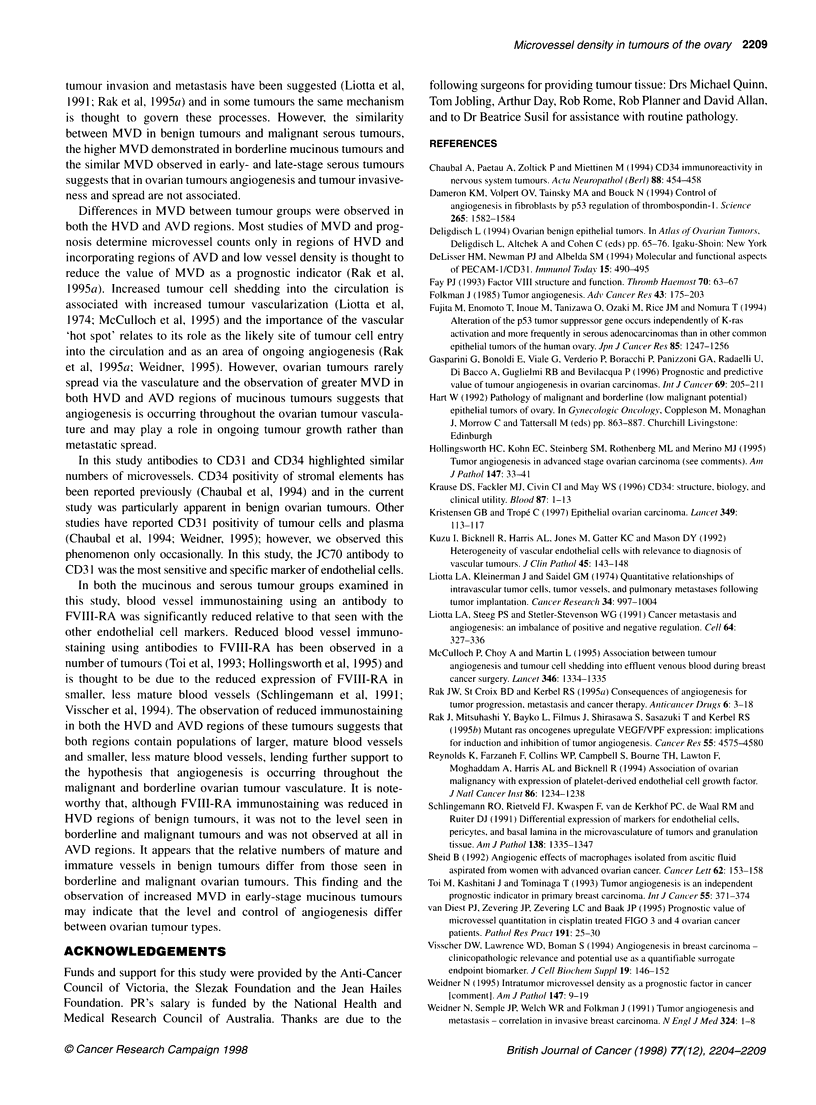# Increased microvessel density in mucinous compared with malignant serous and benign tumours of the ovary.

**DOI:** 10.1038/bjc.1998.367

**Published:** 1998-06

**Authors:** M. Orre, M. Lotfi-Miri, P. Mamers, P. A. Rogers

**Affiliations:** Monash University Department of Obstetrics and Gynaecology Monash Medical Centre, Clayton, Victoria, Australia.

## Abstract

**Images:**


*British Journal of Cancer* (1998) **77**(12), 2204–2209
© 1998 Cancer Research Campaign

### Increased microvessel density in mucinous compared with malignant serous and benign tumours of the ovary

**M Orre, M Lotfi-Miri, P Mamers and PAW Rogers**

Monash University Department of Obstetrics and Gynaecology Monash Medical Centre, 246 Clayton Rd, Clayton, Victoria, 3168, Australia

**Summary** Microvessel density of benign, borderline and malignant ovarian tumours was studied immunohistochemically using antibodies to the endothelial cell markers CD31, CD34 and factor VIII-related antigen. Microvessel density was compared in tumours of different histological subtype, stage and patient outcome. CD31-immunostained sections were examined and regions of high and average microvessel density were selected. Identical regions were located on CD34- and factor VIII-related antigen-immunostained serial sections and microvessel counts obtained and converted to vessels $mm^{-2}$. CD31 and CD34 immunostaining revealed increased microvessel density in both the high and average vessel density regions of mucinous (222.4 ± 24.8; 79.9 ± 8.5) compared with serous (105.4 ± 20.7; 33.3 ± 6.8) and benign (84.4 ± 19.4; 20.4 ± 4.4) tumours ($P < 0.001$). CD31 and CD34 immunostaining also revealed increased microvessel density in early-stage mucinous tumours (234.6 ± 28.2; 87.8 ± 9.2) compared with that observed in both early- (72.8 ± 15; 12.9 ± 2.4) and late- (115.6 ± 26.5; 29.8 ± 8.5) stage serous tumours ($P < 0.001$). No differences in microvessel density in samples from patients with differing outcomes were observed ($P > 0.05$). Reduced factor VIII-related antigen compared with CD31 and CD34 immunostaining was observed in both borderline and malignant mucinous and serous tumours ($P < 0.02$) but not in benign tumours ($P > 0.05$). Our results contradict the putative association between increased microvessel density and poor prognosis and suggest that the level and control of angiogenesis may differ between ovarian tumour types.

**Keywords:** microvessel density; ovarian cancer; mucinous; serous; benign; angiogenesis

Epithelial ovarian cancer is a common gynaecological malignancy. Most patients are diagnosed in the advanced stages of the disease and 5-year survival rates are, at approximately 20%, quite low (Kristensen and Tropé, 1997). Angiogenesis, or the growth of new blood vessels, is essential for the development, growth and spread of solid tumours (Folkman, 1985). Measurements of microvessel density (MVD) are often used to indicate tumour angiogenesis, and two of three recent studies of MVD in ovarian carcinoma have demonstrated an association between increased MVD and reduced patient survival (Hollingsworth et al, 1995; van Diest et al, 1995; Gasparini et al, 1995).

Most ovarian neoplasms arise from the epithelium of the ovary and may be benign, malignant or borderline (low malignant potential tumours). Epithelial ovarian tumours are staged (I–IV) according to tumour spread, and a number of histological subtypes exist, including serous, mucinous, endometrioid, clear cell, transitional cell and mixed epithelial (Kristensen and Tropé, 1997). In this study we quantified MVD in benign tumours and in borderline and malignant tumours of the serous and mucinous types. Malignant serous tumours are generally diagnosed at later stages and survival rates are low. Mucinous tumours are generally early-stage, borderline tumours and patients diagnosed with these tumours have a more favourable outcome (Kristensen and Tropé, 1997). The first aim of this study was to compare microvessel density in benign, mucinous and serous tumours and to relate this to the prognosis of these tumour types.

An association between elevated MVD and patient prognosis has been demonstrated in many tumour types, although other studies have failed to repeat these observations (reviewed in Weidner, 1995). Heterogeneity of the tumour vasculature is well recognized (Weidner et al, 1991; Visscher et al, 1994) and most prognostic studies evaluate MVD in regions of high microvessel density or areas termed vascular 'hotspots'. These regions are not only thought to represent regions of ongoing tumour angiogenesis but also to be the site of tumour cell entry into the circulation (Rak et al, 1995a; Weidner, 1995). However, rather than spreading via the vasculature, ovarian tumours generally spread by peritoneal dissemination (Kristensen and Tropé, 1997) and tumour angiogenesis is unlikely to play a role in this type of spread. The growth of the primary ovarian tumour and its peritoneal metastases is dependent on continued blood vessel growth (Sheid, 1992), and regions other than vascular 'hotspots' may contribute to this growth. Thus, the second aim of this study was to compare MVD in both high vessel density (HVD) and average vessel density (AVD) regions of ovarian tumours of differing prognosis.

A variety of endothelial cell markers can be used to highlight tumour blood vessels immunohistochemically. The most commonly used antibodies include those to factor VIII-related antigen, CD31 and CD34. Factor VIII forms part of the von Willebrand factor complex and plays a role in the coagulation process (Fay, 1993). CD31, also known as platelet-endothelial cell adhesion molecule, is a transmembrane glycoprotein involved in cell–cell adhesion (DeLisser et al, 1994) and CD34 is a surface glycoprotein of unknown function (Krause et al, 1996). The relative abilities of these antibodies to highlight the vasculature has been examined in a number of tumours (Kuzu et al, 1992; Toi et al, 1993), and the final aim of our study was to compare the microvessel staining abilities of these commonly used endothelial cell markers in ovarian tumours.

### MATERIALS AND METHODS

### Tissue specimens

Specimens from benign, malignant and borderline primary ovarian tumours were assessed. Only one block from each tumour was

*Received 11 June 1997*
*Revised 12 December 1997*
*Accepted 6 January 1998*

*Correspondence to:* M Orre

**Table 1** Clinical information for subjects included in this study

| Histological type | n | Age (years) Mean (range) | Clinical stage | | | | Patient outcome | | |
|---|---|---|---|---|---|---|---|---|---|
| | | | I | II | III | IV | Deceased | Recurred | Clear |
| Malignant | | | | | | | | | |
| Serous | 16 | 63 (48–78) | 0 | 1 | 14 | 1 | 9 | 4 | 3 |
| Mucinous | 4 | 59 (41–74) | 3 | 0 | 1 | 0 | 2 | 0 | 2 |
| Borderline | | | | | | | | | |
| Serous | 5 | 45 (29–55) | 4 | 1 | 0 | 0 | 1 | 0 | 4 |
| Mucinous | 12 | 56 (34–87) | 10 | 0 | 2 | 0 | 1 | 1 | 10 |
| Benign | | 54 (23–85) | | | | | | | |
| Cystadenoma | 9 | | – | – | – | – | – | – | – |
| Other | 10 | | – | – | – | – | – | – | – |

available for microvessel quantification. The histopathology of each tumour was confirmed before inclusion in the study. Blocks lacking tumour components or microvessel immunostaining were excluded from the study. A breakdown of the 19 benign and 37 borderline and malignant tumours included in the study and relevant patient characteristics are presented in Table 1. Preliminary follow-up information was collected up to 3 years (average of 21 months) after initial surgery. Staging of the malignant tumours was performed according to International Federation of Gynaecologists and Obstetricians guidelines. Ethical approval for this study was obtained from the Monash Medical Centre Research and Human Ethics Committee.

### Immunohistochemistry

Specimens were fixed in buffered formalin and embedded in paraffin. Serial sections were immunostained using antibodies to the endothelial cell markers CD31 (mouse monoclonal JC/70A; Dako, Botany, NSW, Australia; 1:50), CD34 (mouse monoclonal QBend/10; Serotec, Australian Laboratory Services, Melbourne, Victoria, Australia; 1:40) and factor VIII-related antigen (FVIII-RA) (rabbit polyclonal; Behring, Behring Diagnostics, La Jolla, CA, USA; 1:400). A positive control slide known to exhibit endothelial cell staining with all three markers was included in each staining run. A negative control slide, in which the primary antibody was replaced by the same concentration of the appropriate mouse IgG (CD31 and CD34) or normal rabbit serum (FVIII-RA), was also included in each staining run. Paraffin-embedded serial sections (5 μm) were mounted on 3-aminopropyltriethoxysilane (Sigma, Castle Hill, NSW, Australia)-coated slides, dewaxed and rehydrated. Sections stained using CD31 antibody were predigested for 40 min at 37°C with 1 mg $ml^{-1}$ pepsin (Sigma) in 3% acetic acid. Before incubation with the primary antibody, all sections were quenched for 10 min with 3% hydrogen peroxide in methanol. Non-specific binding of CD31 and CD34 antibody was blocked with a 20-min incubation in 10% normal rabbit serum. Primary antibody was diluted in 1% bovine serum albumin (CD31 and CD34) or 10% fetal calf serum (FVIII-RA) in phosphate-buffered saline (PBS). CD31-immmunostained sections were incubated overnight at room temperature, CD34-stained sections for 45 min at 37°C and FVIII-RA-stained sections for 60 min at 37°C. Following washing with PBS, sections were incubated with an appropriate biotinylated secondary antibody (Zymed, Bioscientific, Melbourne, Victoria, Australia), washed with PBS again and incubated with horseradish peroxidase-conjugated streptavidin (Zymed). After washing with PBS, the colour was developed by incubating the sections with 3-amino-9-ethylcarbazole (Zymed) for 10 min. Sections were then washed with water, counterstained with haematoxylin and mounted with Clearmount (Zymed) mounting medium.

### Microvessel quantification

Sections were analysed using a Zeiss Axioskop microscope, and the images projected to a Sony PVM1440QM video monitor using a Sony CCDIRIS video camera. A preliminary examination of the slides indicated that CD31 antibody gave the most sensitive and specific staining of endothelial cells. In contrast, stromal elements of some tumours were CD34 positive and FVIII-RA antibody appeared to stain fewer microvessels. Therefore sections stained for CD31 were used to select HVD and AVD regions. The sections were scanned at low power to locate regions of high MVD and counts were obtained for these regions (100× magnification, 0.29 $mm^2$ field size). The region with the highest microvessel count was selected and its location within the section noted. The average vessel density region was selected by scanning the section (100 × magnification) and obtaining microvessel counts for up to 20 fields. The average number of vessels per field was calculated and a region representative of this average was selected. Vessels were defined as any positively stained single cell or cluster of cells. The presence of a lumen was not a defining characteristic. The same region on serial sections stained for CD34 and FVIII-RA was located and microvessel counts obtained. Vessel counts per field were converted to vessels $mm^{-2}$.

### Statistics

All statistics were performed using the SPSS statistics package (V6.1.2; SPSS Australasia, North Sydney, NSW, Australia). Vessel density measurements were transformed to obtain homogeneity of variance if required and ANOVA conducted. Multiple comparisons were performed using the Bonferroni *t*-procedure. For single comparisons the data were transformed appropriately and Student's *t*-test performed. The criterion of statistical significance applied was $P < 0.05$.

### RESULTS

### Qualitative differences in blood vessel immunostaining

Vessel density within each tumour section was heterogeneous, readily allowing HVD and AVD regions to be distinguished from each other and from relatively avascular regions. This was evident

© Cancer Research Campaign 1998

**Figure 1** Serial sections of a borderline serous tumour immunostained for CD31 (**A**), CD34 (**B**) and FVIII-RA (**C**). Fewer vessels (indicated by arrowheads) are FVIII-RA positive. CD31-positive vessels in the high (**D**) and average (**E**) vessel density regions of a borderline mucinous tumour are shown. CD31-immunostained section from a late stage serous tumour (**F**) showing both diffuse endothelial cell staining and staining localized to the endothelial cell boundary. Scale bar indicates 50 μm (× 400 magnification)

with all vessel markers tested in this study despite differences in their endothelial cell staining ability (Figure 1). IgG and rabbit serum negative control slides were clear of immunostaining.

Differences in the nature of the staining seen with each antibody were apparent. CD34 staining was generally localized to endothelial cells; however, stromal staining was observed in some samples, particularly those from benign tumours. FVIII-RA staining was frequently of a granular appearance. In some of the more vascular malignant tumours and in the high vessel density regions of some of the less vascular tumours CD31 appeared to be localized to the endothelial cell boundary, giving the vessels a striped appearance (Figure 1). This staining pattern was seen less frequently in benign tumours. In other regions CD31 had a similar diffuse endothelial cell staining pattern to that seen with CD34.

### MVD in mucinous, serous and benign ovarian tumours

The mucinous and serous groups consisted of both borderline and frankly malignant tumours of early and late stage. Analysis of CD31 immunostaining indicated that MVD did not differ between borderline and malignant mucinous tumours in both HVD (238.3 ± 32.1; 204.8±30.1; *t*-test; $P > 0.05$) and AVD (81.9 ± 11.2;

*© Cancer Research Campaign 1998*

**Figure 2** Mean ± s.e.m. vessels mm$^{-2}$ determined using an antibody to CD31 in high (empty bar) and in average (striped bar) vessel density regions are shown. Tumour type is indicated below the chart. *Significantly greater MVD was observed in mucinous relative to serous or benign tumours (ANOVA; $P < 0.001$; d.f. = 2,53)

**Figure 3** Mean ± s.e.m. vessels mm$^{-2}$ determined using an antibody to CD31 in high (empty bar) and in average (striped bar) vessel density regions are shown. Tumour stage and type are indicated below the chart. *Significantly greater MVD was observed in early-stage mucinous relative to early- and late-stage serous tumours (ANOVA; $P < 0.001$; d.f. = 2,53)

74.8 ± 17.4; *t*-test; $P > 0.05$) regions. In HVD regions MVD did not differ (*t*-test; $P > 0.05$) between borderline (58.5 ± 12.2) and malignant (120 ± 26.2) serous tumours. However in AVD regions MVD in borderline tumours (12.9 ± 2.4) was significantly less (*t*-test; $P < 0.01$) than that observed in malignant (40 ± 8.4) tumours. For comparison of histological type, all mucinous tumours and all serous tumours formed two groups. CD31 (Figure 2, Table 2) and CD34 (Table 2) immunostaining revealed significantly greater MVD in both the HVD and AVD regions in mucinous tumours compared with serous tumours and benign tumours (ANOVA; $P < 0.001$; d.f. = 2, 53). Analysis of FVIII-RA immunostaining revealed significantly greater MVD in the HVD regions of mucinous relative to serous malignant tumours (Table 2; ANOVA; $P < 0.05$; d.f. = 2, 53). However, using this antibody, MVD in AVD regions did not differ between histological types (Table 2; ANOVA; $P > 0.05$). In both the HVD and AVD regions, MVD, indicated by any of the three endothelial cell markers, did not differ between malignant serous and benign tumours (Table 2; ANOVA; $P > 0.05$).

### MVD in early- and late-stage ovarian tumours

For comparisons based on tumour stage the histological subtypes remained separate. However, the borderline and malignant groups were combined. Both CD31 (Figure 3, Table 2) and CD34 (Table 2) immunostaining demonstrated significantly greater MVD in the HVD and AVD regions of early-stage mucinous tumours compared with both early- and late-stage serous tumours (ANOVA; $P < 0.001$; d.f. = 2, 31). MVD did not differ between early- and late-stage serous tumours ($P > 0.05$). FVIII-RA immunostaining in HVD regions was significantly greater in early-stage mucinous than late-stage serous tumours (Table 2; ANOVA; $P < 0.05$; d.f. = 2, 31). However, similarly to the comparison of histological type, AVD did not differ between tumour stage when evaluated using FVIII-RA antibody (Table 2; ANOVA, $P > 0.05$; d.f. = 2, 31).

### MVD and patient outcome

Most of the patients diagnosed with mucinous tumours remain clear of disease, allowing analysis of patient outcome and MVD to

**Table 2** Microvessel density in high (HVD) and average (AVD) vessel density regions,assessed using three endothelial cell markers

| Tumour type | Endothelial cell marker: CD1 | | CD34 | | FVIII-RA | |
|---|---|---|---|---|---|---|
| | HVD | AVD | HVD | AVD | HVD | AVD |
| Mucinous | | | | | | |
| All | 222.4 ± 24.8 | 79.9 ± 8.5 | 236.3 ± 28.2 | 103.0 ± 11.9 | 68.0 ± 17.3[b] | 28.9 ± 6.8[b] |
| Stages I–II | 234.6 ± 28.2 | 87.8 ± 9.2 | 250.2 ± 32 | 112.9 ± 12.9 | 75.1 ± 20.4[b] | 28.2 ± 8.2[b] |
| Serous | | | | | | |
| All | 105.4 ± 20.7 | 33.3 ± 6.8 | 85.3 ± 22.4 | 42.8 ± 8.8 | 28.9 ± 7.1[a] | 11.6 ± 2.4 |
| Stages I–II | 72.8 ± 15 | 12.9 ± 2.4 | 36.0 ± 14.6 | 19.7 ± 5.4 | 23.8 ± 8.8[a] | 12.9 ± 5.4[b] |
| Stages III–IV | 115.6 ± 26.5 | 29.8 ± 8.5 | 100.6 ± 27.9 | 50 ± 10.9 | 30.3 ± 8.5* | 11.2 ± 2.4* |
| Benign | 84.4 ± 19.4 | 20.4 ± 4.4 | 83.3 ± 23.5 | 36.0 ± 7.8 | 40.5 ± 10.9 | 21.4 ± 3.7 |

MVD given as vessels mm$^{-2}$ (mean ± s.e.m.). [a]FVIII-RA immunostaining is significantly less than that seen with DC31 and CD 34 (ANOVA; $P < 0.02$);b FVIII-RA immunostaining is significantly less than that seen with CD31 (ANOVA; $P < 0.001$).

© Cancer Research Campaign 1998

**Figure 4** Mean ± s.e.m. vessel $mm^{-2}$ determined using an antibody to CD31 in high (empty bar) and in average (striped bar) vessel density regions are shown. From left to right groups of bars indicate vessel densities in samples from deceased, recurred and clear patients. MVD did not differ with patient outcome ($P < 0.05$)

be conducted using the malignant serous group only. MVD evaluated using CD31 (Figure 4), CD34 and FVIII-RA (data not shown) immunostaining did not differ, in either the HVD or AVD regions, between specimens from patients clear of disease, with recurrent disease or deceased patients (ANOVA; $P > 0.05$; d.f. = 2, 20).

### Comparison of CD31, CD34 and FVIII-RA immunostaining

In the HVD and AVD regions of mucinous tumours antibody to FVIII-RA consistently stained fewer microvessels than antibody to CD31 and CD34 (ANOVA; $P < 0.0001$; Table 2). In serous tumours, reduced FVIII-RA compared with CD31 immunostaining was most apparent in late-stage tumours (ANOVA; $P < 0.02$; Table 2). Although reduced, FVIII-RA immunostaining in benign tumours was not significantly different from that obtained with antibodies to CD31 and CD34 (ANOVA; $P > 0.05$; Table 2).

### DISCUSSION

In this study, we examined MVD in both HVD and AVD regions of ovarian tumours and observed significantly greater MVD in mucinous tumours relative to both serous and benign tumours. Increased MVD in mucinous tumours has been reported previously (Gasparini et al, 1996); however, in this study most of the mucinous tumours were late-stage tumours, and the increased MVD observed was thought to be associated with the poorer prognosis of this tumour type relative to other histological types. In the current study, however, most of the mucinous tumours were early-stage borderline tumours, a group with a good prognosis. Thus, the observation of increased MVD in mucinous tumours relative to frankly malignant and borderline serous tumours seems to contradict the putative association between increased MVD and poor prognosis in ovarian carcinoma (Hollingsworth et al, 1995; Gasparini et al, 1996).

The genetic profiles of malignant and borderline mucinous and serous tumour differ. Mutation of the oncogene K-*ras* has been shown to up-regulate expression of the angiogenic factor vascular endothelial growth factor (Rak et al, 1995*b*). Although mutation of this gene is frequently observed in mucinous tumours, it rarely occurs in serous ovarian tumours (Fujita et al, 1994). In contrast, mutation of the tumour-suppressor gene p53 is more frequently observed in serous ovarian tumours (Fujita et al, 1994) and may be associated with down-regulation of the angiogenesis inhibitor thrombospondin-1 (Dameron et al, 1994). These observations suggest that mucinous and serous ovarian tumours are characterized by different angiogenic pathways. Furthermore, mucinous tumours are generally larger than malignant serous tumours (Hart, 1992), a factor that may also be associated with the increased MVD observed in this tumour type.

When compared by tumour stage, early-stage mucinous tumours exhibited higher MVD relative to both early- and late-stage serous tumours. Because of the small group size, late-stage mucinous tumours were excluded from this section of the study. Despite the poor prognosis of late-stage compared with early-stage serous tumours, no difference in MVD between the two groups was observed. Previous studies of MVD in ovarian tumours have not compared early- and late-stage serous tumours. A recent study of angiogenic growth factor expression in ovarian tumours did not demonstrate any association between pathological stage and expression of platelet-derived endothelial cell growth factor (Reynolds et al, 1994). Although the aggressiveness and pattern of spread of early- and late-stage serous tumours differs, their angiogenic capacity appears to be similar. Patients diagnosed with early-stage mucinous tumours have a good prognosis, and the observation of increased MVD in this group casts further doubt on the putative association between increased MVD and poor prognosis in ovarian tumours.

Comparison of MVD in serous tumour samples from patients with different outcomes did not reveal any differences in MVD. Previous studies examining MVD in ovarian carcinoma have used survival curves to assess the prognostic ability of MVD, and two of these three studies demonstrated an association between higher MVD and reduced survival (Hollingsworth et al, 1995; van Diest et al, 1995; Gasparini et al, 1996). Small group numbers and shorter follow-up times precluded the use of this technique in the current study. As an alternative, we compared MVD in serous tumour samples from deceased patients, those with ongoing disease and those clear of disease and in tumour types with known differences in prognosis. The lack of observed difference between serous tumours with different outcomes may be due to the inclusion of early-stage tumours and the short follow-up times relative to previous studies. It is possible that the use of MVD as a prognostic indicator in ovarian tumours is only applicable to late-stage tumours, a group in which haematogenous spread may occur (Kristensen and Tropé, 1997). In the previous studies of MVD in ovarian tumours, in which an association between increased MVD and reduced survival has been demonstrated (Gasparini et al, 1996; Hollingsworth et al, 1995), most of the tumours have been of the late-stage serous type.

In the current study MVD in benign ovarian tumours was similar to that seen in malignant serous tumours, suggesting that a similar level of angiogenesis is occurring in these two tumour groups. Microvessel density in benign ovarian tumours has not been examined previously; however, these tumours can attain quite large sizes (Deligdisch, 1994) and would be expected to induce an angiogenic response to accommodate this growth. Expression of both VEGF and PDECGF has been demonstrated in benign ovarian tumours (Reynolds et al, 1994). Parallels between tumour angiogenesis,

 © Cancer Research Campaign 1998

tumour invasion and metastasis have been suggested (Liotta et al, 1991; Rak et al, 1995*a*) and in some tumours the same mechanism is thought to govern these processes. However, the similarity between MVD in benign tumours and malignant serous tumours, the higher MVD demonstrated in borderline mucinous tumours and the similar MVD observed in early- and late-stage serous tumours suggests that in ovarian tumours angiogenesis and tumour invasiveness and spread are not associated.

Differences in MVD between tumour groups were observed in both the HVD and AVD regions. Most studies of MVD and prognosis determine microvessel counts only in regions of HVD and incorporating regions of AVD and low vessel density is thought to reduce the value of MVD as a prognostic indicator (Rak et al, 1995*a*). Increased tumour cell shedding into the circulation is associated with increased tumour vascularization (Liotta et al, 1974; McCulloch et al, 1995) and the importance of the vascular 'hot spot' relates to its role as the likely site of tumour cell entry into the circulation and as an area of ongoing angiogenesis (Rak et al, 1995*a*; Weidner, 1995). However, ovarian tumours rarely spread via the vasculature and the observation of greater MVD in both HVD and AVD regions of mucinous tumours suggests that angiogenesis is occurring throughout the ovarian tumour vasculature and may play a role in ongoing tumour growth rather than metastatic spread.

In this study antibodies to CD31 and CD34 highlighted similar numbers of microvessels. CD34 positivity of stromal elements has been reported previously (Chaubal et al, 1994) and in the current study was particularly apparent in benign ovarian tumours. Other studies have reported CD31 positivity of tumour cells and plasma (Chaubal et al, 1994; Weidner, 1995); however, we observed this phenomenon only occasionally. In this study, the JC70 antibody to CD31 was the most sensitive and specific marker of endothelial cells.

In both the mucinous and serous tumour groups examined in this study, blood vessel immunostaining using an antibody to FVIII-RA was significantly reduced relative to that seen with the other endothelial cell markers. Reduced blood vessel immunostaining using antibodies to FVIII-RA has been observed in a number of tumours (Toi et al, 1993; Hollingsworth et al, 1995) and is thought to be due to the reduced expression of FVIII-RA in smaller, less mature blood vessels (Schlingemann et al, 1991; Visscher et al, 1994). The observation of reduced immunostaining in both the HVD and AVD regions of these tumours suggests that both regions contain populations of larger, mature blood vessels and smaller, less mature blood vessels, lending further support to the hypothesis that angiogenesis is occurring throughout the malignant and borderline ovarian tumour vasculature. It is noteworthy that, although FVIII-RA immunostaining was reduced in HVD regions of benign tumours, it was not to the level seen in borderline and malignant tumours and was not observed at all in AVD regions. It appears that the relative numbers of mature and immature vessels in benign tumours differ from those seen in borderline and malignant ovarian tumours. This finding and the observation of increased MVD in early-stage mucinous tumours may indicate that the level and control of angiogenesis differ between ovarian tumour types.

### ACKNOWLEDGEMENTS

Funds and support for this study were provided by the Anti-Cancer Council of Victoria, the Slezak Foundation and the Jean Hailes Foundation. PR's salary is funded by the National Health and Medical Research Council of Australia. Thanks are due to the following surgeons for providing tumour tissue: Drs Michael Quinn, Tom Jobling, Arthur Day, Rob Rome, Rob Planner and David Allan, and to Dr Beatrice Susil for assistance with routine pathology.

### REFERENCES

Chaubal A, Paetau A, Zoltick P and Miettinen M (1994) CD34 immunoreactivity in nervous system tumours. *Acta Neuropathol (Berl)* **88**: 454–458

Dameron KM, Volpert OV, Tainsky MA and Bouck N (1994) Control of angiogenesis in fibroblasts by p53 regulation of thrombospondin-1. *Science* **265**: 1582–1584

Deligdisch L (1994) Ovarian benign epithelial tumors. In *Atlas of Ovarian Tumors*, Deligdisch L, Altchek A and Cohen C (eds) pp. 65–76. Igaku-Shoin: New York

DeLisser HM, Newman PJ and Albelda SM (1994) Molecular and functional aspects of PECAM-1/CD31. *Immunol Today* **15**: 490–495

Fay PJ (1993) Factor VIII structure and function. *Thromb Haemost* **70**: 63–67

Folkman J (1985) Tumor angiogenesis. *Adv Cancer Res* **43**: 175–203

Fujita M, Enomoto T, Inoue M, Tanizawa O, Ozaki M, Rice JM and Nomura T (1994) Alteration of the p53 tumor suppressor gene occurs independently of K-ras activation and more frequently in serous adenocarcinomas than in other common epithelial tumors of the human ovary. *Jpn J Cancer Res* **85**: 1247–1256

Gasparini G, Bonoldi E, Viale G, Verderio P, Boracchi P, Panizzoni GA, Radaelli U, Di Bacco A, Guglielmi RB and Bevilacqua P (1996) Prognostic and predictive value of tumour angiogenesis in ovarian carcinomas. *Int J Cancer* **69**: 205–211

Hart W (1992) Pathology of malignant and borderline (low malignant potential) epithelial tumors of ovary. In *Gynecologic Oncology*, Coppleson M, Monaghan J, Morrow C and Tattersall M (eds) pp. 863–887. Churchill Livingstone: Edinburgh

Hollingsworth HC, Kohn EC, Steinberg SM, Rothenberg ML and Merino MJ (1995) Tumor angiogenesis in advanced stage ovarian carcinoma (see comments). *Am J Pathol* **147**: 33–41

Krause DS, Fackler MJ, Civin CI and May WS (1996) CD34: structure, biology, and clinical utility. *Blood* **87**: 1–13

Kristensen GB and Tropé C (1997) Epithelial ovarian carcinoma. *Lancet* **349**: 113–117

Kuzu I, Bicknell R, Harris AL, Jones M, Gatter KC and Mason DY (1992) Heterogeneity of vascular endothelial cells with relevance to diagnosis of vascular tumours. *J Clin Pathol* **45**: 143–148

Liotta LA, Kleinerman J and Saidel GM (1974) Quantitative relationships of intravascular tumor cells, tumor vessels, and pulmonary metastases following tumor implantation. *Cancer Research* **34**: 997–1004

Liotta LA, Steeg PS and Stetler-Stevenson WG (1991) Cancer metastasis and angiogenesis: an imbalance of positive and negative regulation. *Cell* **64**: 327–336

McCulloch P, Choy A and Martin L (1995) Association between tumour angiogenesis and tumour cell shedding into effluent venous blood during breast cancer surgery. *Lancet* **346**: 1334–1335

Rak JW, St Croix BD and Kerbel RS (1995*a*) Consequences of angiogenesis for tumor progression, metastasis and cancer therapy. *Anticancer Drugs* **6**: 3–18

Rak J, Mitsuhashi Y, Bayko L, Filmus J, Shirasawa S, Sasazuki T and Kerbel RS (1995*b*) Mutant ras oncogenes upregulate VEGF/VPF expression: implications for induction and inhibition of tumor angiogenesis. *Cancer Res* **55**: 4575–4580

Reynolds K, Farzaneh F, Collins WP, Campbell S, Bourne TH, Lawton F, Moghaddam A, Harris AL and Bicknell R (1994) Association of ovarian malignancy with expression of platelet-derived endothelial cell growth factor. *J Natl Cancer Inst* **86**: 1234–1238

Schlingemann RO, Rietveld FJ, Kwaspen F, van de Kerkhof PC, de Waal RM and Ruiter DJ (1991) Differential expression of markers for endothelial cells, pericytes, and basal lamina in the microvasculature of tumors and granulation tissue. *Am J Pathol* **138**: 1335–1347

Sheid B (1992) Angiogenic effects of macrophages isolated from ascitic fluid aspirated from women with advanced ovarian cancer. *Cancer Lett* **62**: 153–158

Toi M, Kashitani J and Tominaga T (1993) Tumor angiogenesis is an independent prognostic indicator in primary breast carcinoma. *Int J Cancer* **55**: 371–374

van Diest PJ, Zevering JP, Zevering LC and Baak JP (1995) Prognostic value of microvessel quantitation in cisplatin treated FIGO 3 and 4 ovarian cancer patients. *Pathol Res Pract* **191**: 25–30

Visscher DW, Lawrence WD, Boman S (1994) Angiogenesis in breast carcinoma – clinicopathologic relevance and potential use as a quantifiable surrogate endpoint biomarker. *J Cell Biochem Suppl* **19**: 146–152

Weidner N (1995) Intratumor microvessel density as a prognostic factor in cancer [comment]. *Am J Pathol* **147**: 9–19

Weidner N, Semple JP, Welch WR and Folkman J (1991) Tumor angiogenesis and metastasis – correlation in invasive breast carcinoma. *N Engl J Med* **324**: 1–8

© Cancer Research Campaign 1998